# Hidden bedside rationing in the Netherlands: a cross-sectional survey among physicians in internal medicine

**DOI:** 10.1186/s12913-021-06229-2

**Published:** 2021-03-16

**Authors:** Ursula W. de Ruijter, Hester F. Lingsma, Willem A. Bax, Johan Legemaate

**Affiliations:** 1 Medical Decision Making Section, Department of Public Health, Erasmus University Medical Center, Rotterdam, The Netherlands; 2 Department of Internal Medicine, Northwest Clinics, Alkmaar, The Netherlands; 3 Health Law Section, Department of Ethics, Law and Humanities, Amsterdam University Medical Centers, location AMC, Amsterdam, The Netherlands

**Keywords:** Healthcare rationing, Bedside rationing, Dutch healthcare system, Choice limitation, Democratic deliberation

## Abstract

**Background:**

Healthcare rationing can be defined as withholding beneficial care for cost reasons. One form in particular, hidden bedside rationing, is problematic because it may result in conflicting loyalties for physicians, unfair inequality among patients and illegitimate distribution of resources. Our aim is to establish whether bedside rationing occurs in the Netherlands, whether it qualifies as hidden and what physician characteristics are associated with its practice.

**Methods:**

Cross-sectional online questionnaire on knowledge of -, experience with -, and opinion on rationing among physicians in internal medicine within the Dutch healthcare system. Multivariable ordinal logistic regression was used to explore relations between hidden bedside rationing and physician characteristics.

**Results:**

The survey was distributed among 1139 physicians across 11 hospitals with a response rate of 18% (*n* = 203). Most participants (*n* = 129; 64%) had experience prescribing a cheaper course of treatment while a more effective but more expensive alternative was available, suggesting bedside rationing. Subsequently, 32 (24%) participants never disclosed this decision to their patient, qualifying it as hidden. The majority of participants (*n* = 153; 75%) rarely discussed treatment cost. Employment at an academic hospital was independently associated with more bedside rationing (OR = 17 95%CI 6.1–48). Furthermore, residents were more likely to disclose rationing to their patients than internists (OR = 3.2, 95%CI 2.1–4.7), while salaried physicians were less likely to do so than physicians in private practice (OR = 0.5, 95%CI 0.4–0.8).

**Conclusion:**

Hidden bedside rationing occurs in the Netherlands: patient choice is on occasion limited with costs as rationale and this is not always disclosed. To what extent distribution of healthcare should include bedside rationing in the Netherlands, or any other country, remains up for debate.

**Supplementary Information:**

The online version contains supplementary material available at 10.1186/s12913-021-06229-2.

## Background

Public healthcare expenditure has been increasing rapidly over the last decades, compromising the economic sustainability of healthcare systems worldwide [1]. Consequently, budget constraints pose poignant allocation questions. One possibility for cost-containment is healthcare rationing. However, rationing can be controversial especially when done at the bedside by physicians.

Defining healthcare rationing has proven difficult. This has resulted in different definitions, complicating international comparison of research [2]. Although some consider healthcare rationing only to be explicit denial of care, others describe it as ‘any implicit or explicit mechanisms that allow people to go without beneficial services’ [2, 3]. Rationing can take place on different levels within a healthcare system [4]. Decisions at the micro level concern individual patients and are often taken by physicians; so-called bedside rationing [5, 6]. Bedside rationing has been defined as ‘the withholding by a physician of a medically beneficial service because of that service’s cost to someone other than the patient’ [5, 7]. Ubel and Goold have proposed three conditions by which to define bedside rationing: “a physician must (1) withhold, withdraw, or fail to recommend a service that, in the physician’s best clinical judgment, is in the patient’s best medical interests; (2) act primarily to promote the financial interests of someone other than the patient (including an organisation, society at large, and the physician himself or herself); and (3) have control over the use of the medically beneficial service” [5, 7]. When bedside rationing is done without revealing the decision to ration or its rationale to the patient, it qualifies as hidden bedside rationing [2, 8].

Hidden bedside rationing can result in unfair inequality and illegitimate distribution of resources [9–12]. Furthermore, it can lead to distribution (or restriction) of resources based on clinically irrelevant characteristics such as ethnicity, gender, age or skin colour [13]. Hidden bedside rationing inherently violates informed consent, as patients consent to a course of treatment whilst insufficiently informed about an existing, more expensive, alternative [8, 14].

Most empirical research into healthcare rationing or, more specifically, bedside rationing, focuses on the willingness to ration or on the factors affecting rationing decisions [15]. A European cross-sectional study estimated that 56.3% of physicians engage in bedside rationing [16]. In the United Kingdom, the NHS rations care on different levels but many decisions are still left to the bedside [17]. Yet little evidence is available regarding the practice of hidden bedside rationing in the Netherlands with its regulated competitive healthcare market. Our objective was to establish whether bedside rationing occurs in the Netherlands, whether it qualifies as hidden and which physician characteristics are associated with its everyday practice.

## Methods

### Study design and participants

All (seven) Dutch academic hospitals were invited to participate, of which one declined. We subsequently invited one large general hospital within the region of each participating academic hospital, of which also one declined. Thus, six academic and five general hospitals participated. We conducted a cross-sectional survey among internists (i.e. consultants) and internal medicine residents (i.e. specialist registrars) employed in these hospitals. All eligible physicians were approached through e-mail with a standardised open invitation to participate (Additional file 1). Participation was voluntary and no reimbursement was offered.

### Setting

The Netherlands has a regulated competitive universal health insurance system since 2006 [18]. Private health insurance is mandatory for all citizens while health insurers are required to cover a statutory benefit package. The government defines the content of this benefit package upon advice of the National Healthcare Institute [18]. The government regulates and subsidises insurance and insurers are not allowed to reject applicants. The vast majority of hospital care is included in the statutory benefit package, but all adults are required to pay an annual maximised deductible (USD 465). In 2015 0,2% of the population was uninsured [18].

The Dutch medical profession includes approximately 20,000 specialists (i.e. consultants) of whom 50% are salaried, 37,5% are in private practice (under fee-for-service or lump-sum) and 12,5% a combination of both [19]. The majority of hospitals are non-profit organisations [18]. Specialists are paid by hospitals, while hospital payment is negotiated between insurers and individual hospitals and is often case-based [18].

Identifying bedside rationing within a healthcare system can be complicated [5, 7]. The conditions proposed by Ubel and Goold entail that opportunities for bedside rationing are potentially abundant in the Dutch healthcare system, as physicians maintain a high degree of control over the use of medically beneficial services [18, 20]. Financial self-interest is negligible for salaried physicians in the Dutch healthcare system. Because balance-billing is not allowed, such financial incentives are also limited for physicians in private practice [18, 20]. However, promoting the financial interest of the employing organisation or society at large remain possible arguments in the Dutch setting [18, 20].

### Definition of variables

Bedside rationing was defined as by Ubel and Goold [5]. Bedside rationing was subsequently considered ‘hidden’ when it complied with two additional conditions: both the decision to set limits as well as the rationale for that decision not being disclosed to patients [12].

Four physician characteristics were hypothesised to influence (hidden) bedside rationing: ‘year of graduation’, ‘residency’, ‘type of employing hospital’ and ‘mode of employment’. Year of graduation and residency were considered because of the possible difference in medical education regarding cost-consciousness as well as the difference in clinical experience. The type of employing hospital (academic or general) was considered, not only because of the differences in caseload but also because of a possible difference in employed physician characteristics. Finally, the mode of compensation has been shown to influence physician behaviour and therefore mode of employment (salaried, private practice or other) was considered as well [21, 22].

### Survey

Because a validated survey on this topic was not available within the Dutch context we established our own. The construction and phrasing of the survey questions was based on a systematic review on the content of previous surveys on this subject [15, 16]. Independent medical professionals were asked to establish face-validity of the survey to properly capture the topic of hidden bedside rationing [23]. A subsequent linguistic evaluation by a question construction expert was performed to exclude ambiguous, confusing or leading questions. After adaptation and a second review by the question construction expert, we pretested the survey by means of a trial run among 20 physicians which disclosed no further methodological, linguistic or practical shortcomings. The choice for a web-survey was made given the ease of distribution and data-collection as well as the fact that it was less prone to socially desirable answers [24]. As rationing has different connotations, the survey specifically addressed the study definitions of both healthcare rationing and bedside rationing [25] (Additional file 2). The survey consisted of four questions regarding everyday practice and four statements regarding physician opinion. Questions used a five-point Likert scale, with a sixth option to give an alternative answer [26, 27]. Because the dimension in question was frequency, answers ranged from never to always [28]. Statements used a seven-point Likert scale with answers ranging from completely disagree [1] to completely agree [7, 26, 27].

### Data collection and analysis

All data were collected through *Explora*, a secure online research tool developed by the Dutch Hospital Association. Descriptive statistics were provided on all data. For statements, Likert scales 1–3 were grouped as ‘disagreed to some extent’ and 5–7 as ‘agreed to some extent’. Missing physician characteristics for completed surveys were imputed using multiple imputation (*n* = 5). Due to the small sample size and unknown distribution of relevant characteristics over the total target population, survey data were not weighted.

**Fig. 1:**
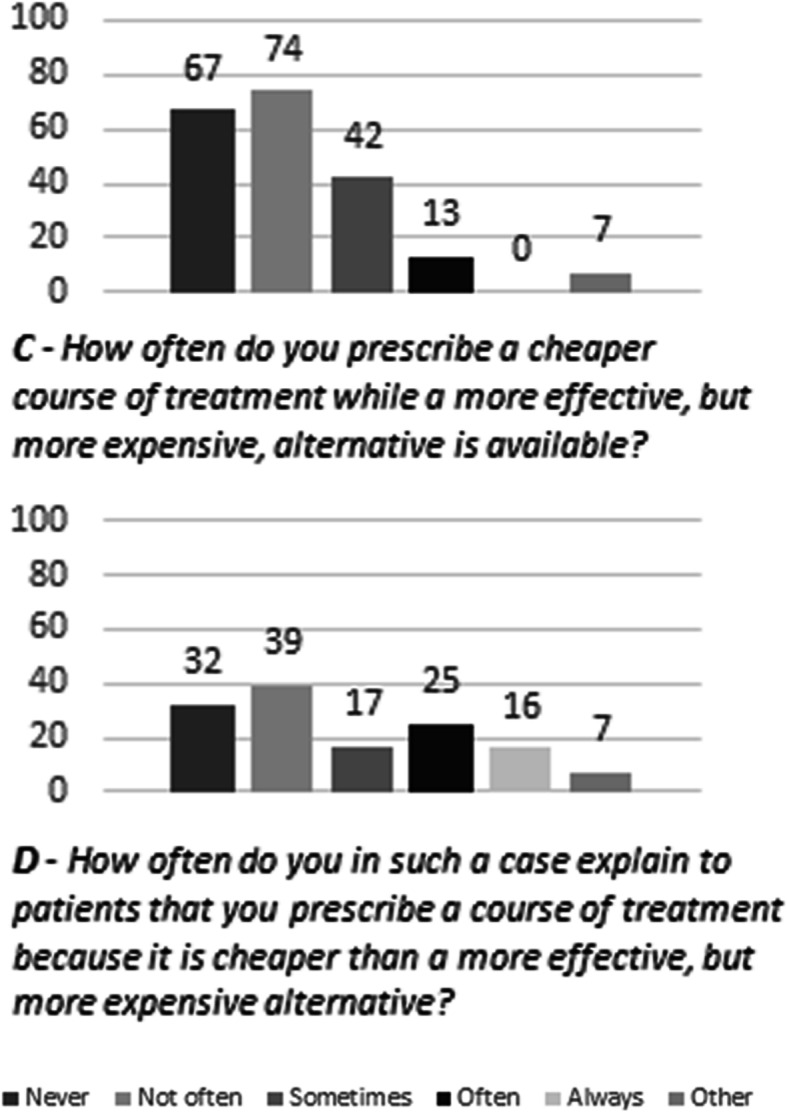
Bar-charts of Outcome for Survey Items on Bedside Rationing and Disclosure Items C-D: Questions regarding everyday practice on a five-point scale ranging from ‘never’ to ‘always’ with a sixth option to give an alternative answer. Item D was answered by a subset of participants (n = 136) who gave a response to item C other than ‘Never’.* The Y-axis depicts number of participants. † Numbers above bars indicate total number of responses per answer

The relationships between the independent variables and bedside rationing, as well as disclosure were explored with univariate and multivariate proportional odds logistic regression for ordinal outcomes. First, we studied bedside rationing by analysing responses to survey item C (Fig. 1, Additional file 2). The answering scale ranged from ‘never’ (i.e. no bedside rationing) to ‘always’ (i.e. bedside rationing). Second, we studied disclosure of bedside rationing by analysing responses to survey item D (Fig. 1, Additional file 2). The answering scale ranged from ‘never’ (i.e. hidden bedside rationing) to ‘always’ (i.e. full disclosure). The effect of the independent variables was expressed as odds ratios and 95% confidence intervals. Subgroup analyses were not planned due to the number of invited participants and limited number of expected participants. We used the STROBE cross sectional checklist when writing our report [29]. All statistical tests were performed in IBM SPSS Statistics 24.

## Results

### Participants

An invitation to participate was sent to 1139 internists and residents. A total of 240 responded (21%) of which 34 respondents did not complete the survey and three were junior doctors. After excluding these, a total of 203 (18%) participants remained of which 86 (42%) were residents and 109 (54%) were employed at an academic hospital (Table 1). With regard to employment mode, 105 (52%) participants indicated to be in training yet only 86 (42%) reported to be a resident. This difference was most likely explained by final year residents working within a fellowship in their respective sub-specialisation and were therefore considered as being in training.

**Table 1:** Summary of Demographic and Employment Characteristics of Survey Participants

Total participants	*n* = 203
Gender^a^	*n (%)*
Female	106 (52)
Not willing to answer	4 (2)
Specialisation^a^	*n (%)*
Acute Internal Medicine	4 (2)
Allergology	1 (1)
Endocrinology	14 (7)
Gastroenterology	5 (3)
Geriatrics	8 (4)
Haematology	8 (4)
Infectious Disease	15 (7)
Intensive Care	5 (3)
Nephrology	18 (9)
Oncology	15 (7)
Vascular Disease	15 (7)
Resident	86 (42)
Not willing to answer	5 (3)
Other	4 (2)
Type of hospital^a^	*n (%)*
Academic hospital	109 (54)
Not willing to answer	1 (1)
Employment mode^a^	*n (%)*
In training	105 (52)
Salaried	49 (24)
Private practice	46 (22)
Not willing to answer	1 (1)
Other	2 (1)
Experience^b^	*mean years (SD)*
Age	39.7 (10.6)
Years since graduation	14.0 (10.1)

^a^All variables except ‘Experience’ are expressed as n (%)

^b^‘Experience’ is expressed as mean number of years (standard deviation)

### Bedside rationing

A total of 88 (43%) participants could envision a physician denying a patient a course of treatment because of cost consideration. When asked about their everyday practice, 129 (64%) participants had prescribed a cheaper course of treatment while a more effective, but more expensive, alternative had been available. Of these physicians, 13 (6%) reported doing so often. Other answers (*n* = 7; 3%) included physicians who disclosed they did not feel informed enough about costs in order to consider it. All physicians with experience prescribing cheaper treatment were subsequently asked how often they disclose this to their patients. Most (*n* = 97; 71%) disclosed this on some occasions. Sixteen participants (12%) reported to always disclose prescribing less effective but cheaper treatment while 32 (24%) indicated to never disclose such decisions (Fig. 1).

Nearly three quarter of participants (*n* = 148; 73%) agreed to some extent that a physician should explain their cost considerations when withholding a course of treatment that is considered too expensive. Fifty-eight participants (29%) were in complete agreement and only four (2%) completely disagreed. Among the additional clarifications, participants agreed that explanation should be provided, but the decision to ration should not be made by physicians in the consulting room but by policymakers in an overarching mechanism.

### Treatment cost

Some physicians reported that lacking knowledge of treatment cost led them to not consider costs when prescribing a course of treatment. Half of participants (*n* = 100; 49%) reported they rarely feel sufficiently informed about treatment cost to discuss it, while 2 % (*n* = 4) always feels equipped to do so. None reported always bringing up treatment cost, compared to 60 participants that reported never to do so (30%). Most indicated they do not discuss it often (*n* = 93; 46%) opposed to three (2%) that bring up treatment cost regularly.

### Physician characteristics affecting bedside rationing

The univariate analysis showed that physicians working in an academic hospital reported more bedside rationing behaviour compared to physicians working in a general hospital (OR = 20.5 95% CI 15.4–27.3, *p* < 0.001). Residents reported more bedside rationing behaviour compared to internists (OR = 10.5 95% CI 8.1–13.5, *p* < 0.001). In the multivariable analysis only ‘type of hospital’ had a statistically significant effect. Physicians working in an academic hospital reported a higher frequency of bedside rationing behaviour compared to physicians working in a general hospital (OR = 17.0 95% CI 6.1–47.9, *p* < 0.001) (Table 2).

**Table 2:** Ordinal Regression Analysis on Physician Characteristics Affecting Bedside Rationing

	Univariate analysis			Multivariate analysis		
	OR	95% CI	*p*	OR	95% CI	*p*
Years Since Graduation (per 10 years)	1.1	1.0–1.2	0.10	0.9	0.6–1.3	0.49
Residency	**10.5**	**8.1–13.5**	**< 0.001**	1.8	0.7–4.4	0.37
Academic Hospital	**20.5**	**15.4–27.3**	**< 0.001**	**17.0**	**6.1–47.9**	**< 0.001**
Employment Mode						
Private Practice	1.0	(ref)		1.0	(ref)	
Salaried	0.8	0.6–1.1	0.13	0.6	0.2–1.2	0.16
In Training	0.9	0.7–1.1	0.26	0.8	0.3–2.0	0.58

Dependent variable is response to item C in the survey on a five-point Likert scale ranging from ‘never’ (i.e. no bedside rationing) to ‘always’ (i.e. bedside rationing). Variables are expressed as odds ratios with 95% confidence intervals. Statistically significant effects are expressed in bold

### Physician characteristics affecting disclosure of bedside rationing

The univariate analysis showed that residents reported more disclosure of bedside rationing to patients compared to internists (OR = 23.9 95% CI 16.7–34.3, *p* < 0.001). An increase in years since graduation (per 10 years) was associated with an increase in the odds of disclosure (OR = 1.7 95% CI 1.5–1.9, *p* < 0.001). In the multivariate analysis ‘residency’, ‘mode of employment’, ‘mode of payment and ‘years since graduation (per 10 years)’ had a statistically significant effect on disclosure. An increase in years since graduation (per 10 years) was associated with an increase in the odds of disclosure, with an odds ratio of 1.5 (95% CI 1.2–1.8, *p* < 0.001). Residents reported more frequent disclosure compared to internists (OR = 3.2 95% CI 2.1–4.7, *p* < 0.001). Salaried physicians reported less frequent disclosure compared to physicians in private practice (OR = 0.5 95% CI 0.4–0.8, *p* = .002). Similarly, physicians in training reported less frequent disclosure compared to physicians in private practice (OR = 0.5 95% CI 0.3–0.9, *p* = .014) (Table 3).

**Table 3:** Ordinal Regression Analysis on Physician Characteristics Affecting Disclosure of Bedside Rationing

	Univariate analysis			Multivariate analysis		
	OR	95% CI	*p*	OR	95% CI	*p*
Years Since Graduation (per 10 years)	**1.7**	**1.5–1.9**	**< 0.001**	**1.5**	**1.2–1.8**	**< 0.001**
Residency	**23.9**	**16.7–34.3**	**< 0.001**	**3.2**	**2.1–4.7**	**< 0.001**
Academic Hospital	0.0	0.0–0.0	1.00	0.0	0.0–0.0	1.00
Employment Mode						
Private Practice	1.0	(ref)		1.0	(ref)	
Salaried	1.0	0.7–1.4	0.98	**0.5**	**0.4–0.8**	**0.002**
In Training	**0.4**	**0.3–0.5**	**< 0.001**	**0.5**	**0.3–0.9**	**0.01**

Dependent variable is response to item D in the survey on a five-point Likert scale ranging from ‘never’ (i.e. hidden bedside rationing) to ‘always’ (i.e. full disclosure). Variables are expressed as odds ratios with 95% confidence intervals. Statistically significant effects are expressed in bold

## Discussion

The objective of this study was to establish whether bedside rationing occurs in the Netherlands, whether it qualifies as hidden and whether there are associations with specific physician characteristics. To our knowledge, this is the first empirical study that explores physicians’ knowledge of and experience with bedside rationing in the Netherlands. We found that physicians occasionally limit patient choice for reasons of cost, indicating that bedside rationing indeed occurs. Furthermore, we found that the decision to set these limits and its rationale are not always disclosed to the patient, qualifying it as hidden. Nearly two thirds of participating physicians indicate they have at some point engaged in bedside rationing. Although the occurrence of bedside rationing is in line with previous evidence, the extent is larger than expected [16, 30]. Residency and working at an academic hospital were associated with more bedside rationing. On the other hand, residents are more likely to disclose bedside rationing decisions to their patients than internists while salaried physicians are less likely to do so than physicians who are in private practice. Hidden bedside rationing in the Netherlands therefore seems to be more prevalent among salaried internists. As described previously, salaried physicians have negligible financial self-interest. However, there are myriad incentives for bedside rationing other than self-interest such as organisational structures, financial incentives for the employer or even society at large. Further (qualitative) research is needed to provide answers on the underlying mechanisms.

The consequences of hidden bedside rationing are potentially severe [9, 10, 13]. Most physicians acknowledged this and commented they do not consider the bedside the appropriate setting for rationing decisions. The wish for such decisions to be made away from the bedside is not new. For example, 70% of randomly selected neurologists in the US agreed that if rationing decisions have to be made, they should be made away from the bedside and specifically not while practitioners are caring for patients [31].

Our findings can therefore be taken as another argument in favour of a more explicit alternative to bedside rationing. One such alternative is administrative gatekeeping: a concept which is an intermediate between unrestricted advocacy of patients and bedside rationing [12]. Physicians are still involved in rationing, but at higher organisational levels. It allocates the conflicting duties to different locations: unrestricted patient advocacy at the bedside and rationing scarce resources when constructing (clinical) guidelines and policy. In a study of randomly selected GPs 69% agreed that physicians should adhere to guidelines that discourage the use of expensive interventions with a small advantage over (cheaper) standard interventions [16]. This would entail that physicians at times have to provide less than optimal treatment to individual patients when this results from the agreed upon policy.

Although the potential for unfair inequality and illegitimate distribution of resources as a result of hidden bedside rationing warrants a more explicit way of rationing, some degree of bedside rationing is unavoidable [10, 12, 32]. Priority setting is and will remain an important part of healthcare policy. A more explicit way of rationing has its own drawbacks. Decisions at higher organizational levels are imprecise and aimed at large groups of patients rather than tailored to individuals [33]. Moreover, it may add to an already much debated-upon administrative burden for physicians, both in the Netherlands and abroad [34, 35]. One aim of a more explicit system is to improve transparency in decision-making in order to increase the willingness of both the public and other stakeholders to accept rationing decisions [36, 37]. Yet, it is the perception of a transparent decision-making process rather than actual transparency that increases acceptance of decisions [36, 37]. Research into explicit alternatives therefore needs to address their potential impact on bureaucracy, inappropriate care and stakeholder perception as these could be barriers in a more explicit system.

Another notable finding is that three quarter of participants rarely discuss treatment cost with patients in their everyday practice, of which around a third even reports never to do so. However, when asked for their opinion, nearly three quarters believe a physician ought to explain this decision and its (cost) reasons. The latter is in line with international evidence [33, 38, 39]. For example, 62% of randomly selected US physicians agreed it is important to discuss treatment cost with patients [39]. The discrepancy between everyday practice and physicians’ views is possibly explained in part by the fact that a mere four participants (2%) feel sufficiently informed about treatment cost. These findings tie into research where 59% of physicians indicated that a better knowledge of costs would change the way they order investigations [40]. Rationing decisions at the bedside are therefore made while physicians are insufficiently informed about treatment cost, which in turn raises questions as to how well these decisions are being made [33]. Other factors contributing to failure to discuss treatment cost are likely health system related. In the Netherlands, healthcare included in the statutory benefit package poses no direct financial risk to patients other than the maximised deductible and some specific out-of-pocket payments. Therefore, physicians in the Netherlands might not prioritise informing patients about treatment cost which might be different in other healthcare systems [18, 41].

As previous research also emphasised, there is a need for a more extensive public debate on hidden bedside rationing as well as on its possible (more explicit) alternatives [11, 42, 43]. This debate should include patients and the general public (i.e. future patients), as well as physicians and policymakers. Recently, a citizen panel conducted in the Netherlands systematically asked citizens from various backgrounds about the importance of different criteria to set limits [44, 45]. Building on this and other international studies, future research might focus on rational democratic deliberation as legitimisation of overarching rationing policies [42, 46, 47].

### Limitations

As with previous studies regarding this topic, physicians might have been hesitant to report engaging in bedside rationing due to the sensitivity of the topic. Moreover, self-reporting is limited to bedside rationing that physicians are aware of. Therefore, our survey may underestimate the occurrence of bedside rationing.

The response rate, though in line with previous surveys among medical personnel, is low [48]. Respondents not-missing-at-random may have led to both an over- and an underestimation. It is possible that physicians who are more cost-conscious are more willing to participate and vice versa. Due to the small sample, survey weights were not used and could therefore not account for the non-response. However, participant characteristics are distributed evenly across type of hospital, residency, gender and employment mode.

Our results are limited to one medical specialisation in 11 hospitals in the Dutch healthcare system. The choice for internal medicine is in line with previous research on bedside rationing [15, 16, 49]. Although there is no reason to assume this sample differs greatly from the whole Dutch population of internists, extrapolations to other medical specialisations or healthcare systems ought to be done with caution.

Finally, we cannot conclude cost-reduction is the sole reason for bedside rationing due to the construction of the survey. Additionally, no formal reliability tests were performed to formally assess validity and reliability of the survey other than establishing face validity. In the questions pertaining to prevalence of bedside rationing the reasoning for choice limitation is not phrased explicitly. We can therefore only establish an association rather than causality. Furthermore, the questions are limited to rationing of treatment and therefore do not provide answers on the potential of rationing of other services (e.g. diagnostics, medical consultations or even level of experience of medical personnel). If rationing of services other than treatment indeed occurs, this would entail (hidden) bedside rationing is more widespread than estimated by our study. Moreover, no quantification of ‘more effective’ or ‘more expensive’ is provided in the survey. This is notably difficult yet important: very expensive treatment which is only slightly more effective could be easier to forego than when it would be tremendously more effective.

## Conclusions

Our study indicates that hidden bedside rationing occurs in the Netherlands. Residency and working at an academic hospital are associated with more bedside rationing. Residents are more likely to disclose bedside rationing decisions to their patients than internists while salaried physicians are less likely to do so than physicians who are in private practice. The question of how distribution of healthcare ought to take place and to what degree that should include bedside rationing remains a topic of interest and warrants a more public debate. Embedding informed public and expert opinion into policies at higher organisational levels can decrease the need for bedside rationing.

## Supplementary Information

**Additional file 1.** English Translation of Standardised Open Invitation to Participate.

**Additional file 2.** English Translation of Original Dutch Survey.

**Additional file 3.** Survey Results.

**Additional file 4.** Qualitative Summary of Open Text Answers in English.

## Data Availability

The datasets generated and/or analysed during the current study including all individual deidentified participant data are available from the corresponding author upon reasonable request. An English translation of the survey, its complete results and a qualitative summary of comments or clarifications provided in open text fields are provided as additional files (Additional files 2, 3, 4).

## Abbreviations

MD: Medical Doctor
ORCID: Open Researcher and Contributor ID
PhD: Doctor of Philosophy
NHS: National Health Service (United Kingdom National Health Service)
USD: United States Dollar
STROBE: STrengthening the Reporting of OBservational studies in Epidemiology
IBM: International Business Machines Corporation
SPSS: Statistical Package for the Social Sciences
SD: Standard Deviation
CI: Confidence Interval
US: United States of America
