# Supplementary material for: Hidden bedside rationing in the Netherlands: a cross-sectional survey among physicians in internal medicine
Source: BMC Health Serv Res. 2021 Mar 16;21:233. doi: 10.1186/s12913-021-06229-2 (PMC7967991; doi:10.1186/s12913-021-06229-2)
Supplement: Supplementary file 1 — Additional file 1. English Translation of Standardised Open Invitation to Participate. [file 12913_2021_6229_MOESM1_ESM.docx]

## Additional File 1 - English Translation of Standardised Open Invitation to Participate

Dear Sir / Madam,

Curbing the rise of healthcare expenditure is a topical issue that influences undoubtedly be dealing with. In the Dutch healthcare system, demand already exceeds supply and is only expected to increase in the coming decades. Healthcare will have to be "rationed” as a limited resource. The word rationing in the Dutch language is often associated with shortages, e.g. a food shortage and food stamps. However, in healthcare it can be defined as ‘*any implicit or explicit mechanism that allow patients to go without beneficial services*’. There are of course many different of these mechanisms possible such as in government policy, clinical guidelines or in the prescription of medication.

Yet when rationing enters the doctor’s office and comes into play in the doctor-patient relationship, physicians can be put in a difficult position. In this situation physicians withhold a medically beneficial service from a patient because of that service’s cost to someone other than the patient (such as an organisation or society at large). In our study we focus on rationing decisions by physicians at this individual patient level (i.e. bedside rationing) and the disclosure of these decisions to patients. The objective of our study is to establish whether bedside rationing occurs in the Netherlands, whether it qualifies as ‘hidden’ (i.e. undisclosed) and what physician characteristics are associated with its everyday practice. We therefore would like to ask you to complete an online survey. We have invited the departments of Internal Medicine of all Dutch academic hospitals to participate as well as one general hospital in each academic region.

Completing the survey takes no more than ten minutes. You respond in a secure online environment, which has been developed in collaboration with the Dutch Hospital Association. The data you provide will be processed anonymously.

To start your survey immediately, click on the following [link]. You can start your survey at any time and come back to it at a later date.

If you have any questions or remarks as a result of this email, please do not hesitate to contact us.

Yours sincerely,

[name, email and signature principal investigator]

On behalf of,

[name, email and signature head of department]
