# Supplementary material for: Hidden bedside rationing in the Netherlands: a cross-sectional survey among physicians in internal medicine
Source: BMC Health Serv Res. 2021 Mar 16;21:233. doi: 10.1186/s12913-021-06229-2 (PMC7967991; doi:10.1186/s12913-021-06229-2)
Supplement: Supplementary file 2 — Additional file 2. English Translation of Original Dutch Survey. [file 12913_2021_6229_MOESM2_ESM.docx]

## Additional File 2 - English Translation of Original Dutch Survey

|  | **Welcome**  Thank you very much for your participation in this study. The survey consists of four questions and four statements. Completing this survey will take no more than ten minutes. On the next page you will find a short overview of the used definitions in this study followed by questions pertaining to your everyday experience with bedside rationing and statements concerning your opinion on bedside rationing. At the final page of this survey you have the opportunity to share any remarks. For questions regarding this study you can contact [email principal investigator]. Click on the arrow on the bottom right to start the survey. |
| --- | --- |
|  | **Informed Consent**  Participation in this survey is completely voluntary and no remuneration is offered. Study results will be processed completely anonymously and will be used for scientific research only. All data is stored and processed under current privacy legislation.   - I understand the above and consent to participation in this study |
|  | **Definition**  The term ‘healthcare rationing’ can have different connotations to different people. In this study we define healthcare rationing as any implicit or explicit mechanisms that allow people to go without beneficial services. Rationing decisions at the micro level often concern individual patients and are often made by physicians; so-called bedside rationing. In this study bedside rationing must comply with three conditions: a physician must (1) withhold, withdraw, or fail to recommend a service that, in the physician’s best clinical judgment, is in the patient’s best medical interests; (2) act primarily to promote the financial interests of someone other than the patient (including an organisation, society at large, and the physician himself or herself); and (3) have control over the use of the medically beneficial service. |
| **A** | *This question concerns your experience in everyday practice. It pertains to individual doctor-patient situations and specifically not to situations where expertise is provided for policy or guidelines.*  How often do you feel sufficiently informed about treatment cost in order to be able to discuss this with your patient?   - Never - Not often - Sometimes - Often - Always - Other: [open text field] |
| **B** | *This question concerns your experience in everyday practice. It pertains to individual doctor-patient situations and specifically not to situations where expertise is provided for policy or guidelines.*  How often do you discuss treatment cost with your patient?   - Never - Not often - Sometimes - Often - Always - Other: [open text field] |
| **C** | *This question concerns your experience in everyday practice. It pertains to individual doctor-patient situations and specifically not to situations where expertise is provided for policy or guidelines. It pertains to all courses of treatment that you are at liberty to prescribe. Any course of treatment that is not available to you, either physically or through policy, is therefore beyond the scope of this question.*  How often do you prescribe a cheaper course of treatment while a more effective, but more expensive, alternative is available?   - Never - Not often - Sometimes - Often - Always - Other: [open text field] |
| **D** | *This question concerns your experience in everyday practice. It pertains to individual doctor-patient situations and specifically not to situations where expertise is provided for policy or guidelines. It pertains to all courses of treatment that you are at liberty to prescribe. Any course of treatment that is not available to you, either physically or through policy, is therefore beyond the scope of this question.*  How often do you in such a case explain to patients that you prescribe a course of treatment because it is cheaper than a more effective, but more expensive alternative?   - Never - Not often - Sometimes - Often - Always - Other: [open text field] |
| **E** | *Can you indicate to what extent you agree with the following statement on a scale from 1 (completely disagree) to 7 (completely agree)? This statement pertains to individual doctor-patient situations and specifically not to situations where expertise is provided for policy or guidelines.*  As a physician one carries responsibility to contain healthcare cost  Additional clarification: [open text field] |
| **F** | *Can you indicate to what extent you agree with the following statement on a scale from 1 (completely disagree) to 7 (completely agree)? This statement pertains to individual doctor-patient situations and specifically not to situations where expertise is provided for policy or guidelines.*  If a physician does not prescribe a course of treatment because this is too expensive, he or she ought to explain these cost considerations to the patient  Additional clarification: [open text field] |
| **G** | *Can you indicate to what extent you agree with the following statement on a scale from 1 (completely disagree) to 7 (completely agree)? This statement pertains to individual doctor-patient situations and specifically not to situations where expertise is provided for policy or guidelines.*  I can envision a physician denying a patient a course of treatment because of cost consideration  Additional clarification: [open text field] |
| **H** | *Can you indicate to what extent you agree with the following statement on a scale from 1 (completely disagree) to 7 (completely agree)? This statement pertains to individual doctor-patient situations and specifically not to situations where expertise is provided for policy or guidelines.*  Cost should not play a role in choosing a course of treatment  Additional clarification: [open text field] |
