# Supplementary material for: Hidden bedside rationing in the Netherlands: a cross-sectional survey among physicians in internal medicine
Source: BMC Health Serv Res. 2021 Mar 16;21:233. doi: 10.1186/s12913-021-06229-2 (PMC7967991; doi:10.1186/s12913-021-06229-2)

## Additional File 3 – Survey Results

Additional File 3 Table 1 Distribution of Question Answers Answering scale ranges from Never to Always with a sixth option to give an alternative answer

|  | **Never** | **Not often** | | | **Sometimes** | **Often** | **Always** | | **Other** |
| --- | --- | --- | --- | --- | --- | --- | --- | --- | --- |
| **A** | How often do you feel sufficiently informed about treatment cost in order to be able to discuss this with your patient? | | | | | | | | |
| **% (*n)*** | 7,4% (15) | | 41,9% (85) | 30,5% (62) | | 15,3% (31) | 2,0% (4) | 3,0% (6) | |
| **B** | How often do you discuss treatment cost with your patient? | | | | | | | | |
| **% (*n)*** | 29,6% (60) | 45,8% (93) | | | 22,7% (46) | 1,5% (3) | 0,0% (0) | | 0,5% (1) |
| **C** | How often do you prescribe a cheaper course of treatment while a more effective, but more expensive, alternative is available? | | | | | | | | |
| **% (*n)*** | 33,0% (67) | 36,5% (74) | | | 20,7% (42) | 6,4% (13) | 0,0% (0) | | 3,4% (7) |
| **D** | How often do you in such a case explain to patients that you prescribe a course of treatment because it is cheaper than a more effective, but more expensive alternative? | | | | | | | | |
| **% (*n)*** | 23,5% (32) | 28,7% (39) | | | 12,5% (17) | 18,4% (25) | 11,8% (16) | | 5,1% (7) |

* All variables are expressed as *n* (%)

† Item D was answered by a subset of participants (n=136) who gave a response to item C other than ‘*Never*’.

Additional File 3 Figure 1 Stacked Bar Chart of Question Answers Answering scale ranges from Never to Always


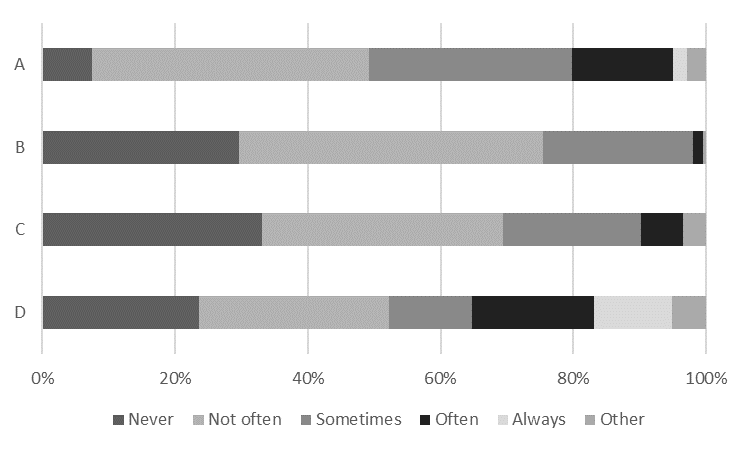


Additional File 3 Table 2 Distribution of Statement Answers Likert Scale ranges from 1 (completely disagree) to 7 (completely agree)

|  | **1** | **2** | **3** | **4** | **5** | **6** | **7** |
| --- | --- | --- | --- | --- | --- | --- | --- |
| **E** | As a physician one carries responsibility to contain healthcare cost | | | | | | |
| *n* | 2 | 7 | 15 | 35 | 77 | 42 | 25 |
| % | 1,0% | 3,4% | 7,4% | 17,2% | 37,9% | 20,7% | 12,3% |
| **F** | If a physician does not prescribe a course of treatment because this is too expensive, he or she ought to explain these cost considerations to the patient | | | | | | |
| *n* | 4 | 17 | 11 | 23 | 38 | 52 | 58 |
| % | 2,0% | 8,4% | 5,4% | 11,3% | 18,7% | 25,6% | 28,6% |
| **G** | I can envision a physician denying a patient a course of treatment because of cost consideration | | | | | | |
| *n* | 22 | 36 | 32 | 25 | 39 | 32 | 17 |
| % | 10,8% | 17,7% | 15,8% | 12,3% | 19,2% | 15,8% | 8,4% |
| **H** | Cost should not play a role in choosing a course of treatment | | | | | | |
| *n* | 32 | 41 | 48 | 22 | 23 | 19 | 18 |
| % | 15,8% | 20,2% | 23,6% | 10,8% | 11,3% | 9,4% | 8,9% |

* All variables are expressed as *n* (%)

Additional File 3 Figure 2 Stacked Bar Chart of Statement Answers Likert scale ranges from 1 (completely disagree) to 7 (completely agree)


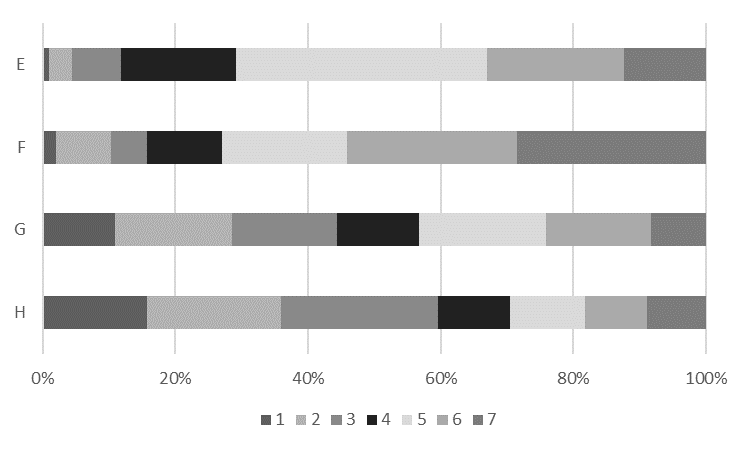

Supplement: Supplementary file 3 — Additional file 3. Survey Results. [file 12913_2021_6229_MOESM3_ESM.docx]
