# Supplementary material for: Hidden bedside rationing in the Netherlands: a cross-sectional survey among physicians in internal medicine
Source: BMC Health Serv Res. 2021 Mar 16;21:233. doi: 10.1186/s12913-021-06229-2 (PMC7967991; doi:10.1186/s12913-021-06229-2)
Supplement: Supplementary file 4 — Additional file 4. Qualitative Summary of Open Text Answers in English. [file 12913_2021_6229_MOESM4_ESM.docx]

## Additional File 4 – Qualitative Summary of Open Text Answers in English

Additional File 4 Table 1 Summary of Open Text Answers by Item

| **Item** | **Answered ‘Other’** | **Item** | **Clarifications** |
| --- | --- | --- | --- |
| Item A | 6 (3%) | Item E | 36 |
| Item B | 1 (0,5%) | Item F | 30 |
| Item C | 12 (6%) | Item G | 29 |
| Item D | 10 (7%) | Item H | 25 |
|  |  |  |  |
| Final comments | 17 |  |  |

* Item variables A-D are expressed as *n* (% of total item answers)

† Item D was answered by a subset of participants (n=136) who gave a response to item C other than ‘*Never*’.

**Item A**

*How often do you feel sufficiently informed about treatment cost in order to be able to discuss this with your patient?*

Other comments: 6

Three physicians commented they rarely inform their patients about treatment cost

Two physicians commented they do not want to enter into a discussion about cost with their patients and do not consider it important to inform their patients about treatment cost

One physician commented they feel insufficiently informed about treatment cost

**Item B**

*How often do you discuss treatment cost with your patient?*

Other comments: 1

One physician answered to only discuss treatment cost when a declaration of use (*Dutch*: *Artsenverklaring*) is required

**Item C**

*How often do you prescribe a cheaper course of treatment while a more effective, but more expensive, alternative is available?*

Other comments: 12

Four physicians answered to be unaware of this or not to know the answer

Two physicians answered to have insufficient knowledge regarding treatment cost

Two physicians answered to regularly choose a cheaper alternative if efficacy was equal or sufficient

Two physicians answered this is a choice they never had to make

One physician answered to never do this because most effective therapy is of sole importance

One physician answered not often and only to do so based on side effects rather than price

**Item D**

*How often do you in such a case explain to patients that you prescribe a course of treatment because it is cheaper than a more effective, but more expensive alternative?*

Other comments: 10

Three physicians answered this is a choice they never had to make

Three physicians answered to only discuss it when they refer to reimbursement limitations or the reimbursement status by the healthcare insurer

One physician answered to never do this because most effective therapy is of sole importance

One physician answered not often and only to do so based on side effects rather than price

One physician answered to have insufficient knowledge regarding treatment cost

One physician answered to always choose a generic when choosing a new treatment

**Item E**

*As a physician one carries responsibility to contain healthcare cost*

Clarifications: 36

Some clarifications contained multiple comments.

Statements emphasising joint responsibility with other stakeholders: 18

Statements emphasising need for rationing decisions to be made outside the consulting room: 14

Statements emphasising the need to reduce unnecessary health care expenses: 10

Statements emphasising the importance of (increased) cost-awareness among patients: 3

Statements emphasising lack of knowledge about cost: 2

Statements emphasising physician is in a better position to allocate care than other stakeholders: 2

Statements emphasising this should not be a responsibility of the physician at all: 1

Statements emphasising cost-containment necessary to keep healthcare accessible for all patients: 1

Statements emphasising individual physicians have little influence on healthcare cost: 1

One physician stated not everything that is technically possible is justified

One physician commented that some treatment choices are not discussed with patients because these are not reimbursed when it would be more just to do so since some might be willing to pay the extra money if they were informed of the option.

One physician emphasised it might be more cost-efficient if general practitioners referred certain patient groups earlier.

One physician commented that healthcare expenditure in hospitals is only part of the total expenditure. There is a responsibility but cost-containment in the consulting room will not solve the whole problem

**Item F**

*If a physician does not prescribe a course of treatment because this is too expensive, he or she ought to explain these cost considerations to the patient*

Clarifications: 30

Some clarifications contained multiple comments.

Statements emphasising the need for rationing decisions to be made outside the consulting room on another level (policymakers, government): 10

Statement emphasising this is not necessary when treatment is equally effective: 6

Statements emphasising this is a difficult topic to discuss and that there is aversion to discussing cost among patients: 6

Statements emphasising a physicians’ primary responsibility is towards their patient: 4

Statement emphasising disclosure harms the patient-doctor relationship: 4

Statement emphasising to always explain treatment choice: 3

Statement emphasising to explain this is only done when it is beyond control of the physician (insurer policy etc.): 3

Statements emphasising there is no time to explain this properly to patients: 3

Statements emphasising to refer to protocol or practice standards in which cost is incorporated but not to refer to cost per se: 2

Statement emphasising the patient’s right to be fully informed about cost: 2

Statements emphasising the physician does not consider it their responsibility to inform patients about treatment cost: 2

Statements emphasising this is only necessary when it is a personal decision rather than one by protocol: 2

One physician stated this only needs to be disclosed when cost is the sole reason. However, it often is a combination of factors. Example given is that of a patient with a bad hip.

One physician stated that patients often feel burdened by expensive courses of treatment

One physician stated it would be good to always start with the cheaper treatment and to only upgrade if the effect is not sufficient

One physician stated that before this can happen patients need to be better educated too

One physician stated ‘too expensive’ is a very broad term

**Item G**

*I can envision a physician denying a patient a course of treatment because of cost consideration*

Clarifications: 29

Some clarifications contained multiple comments.

Statements emphasising cost is not the only consideration: 12

Statements emphasising this is (only) acceptable when the gain is small, and the costs are large: 11

Statements emphasising the difference between life prolonging and life-saving treatment: 4

Statements emphasising the need for rationing decisions to be made by other stakeholders (policymakers, government): 4

Statements emphasising the need for rationing decisions to be made by physicians on another level (hospital policy, national policy): 3

Statements emphasising this is not acceptable when there are no alternatives to the course of treatment: 3

Statements emphasising the importance of these decisions to prevent further opportunity cost or crowding out effects: 3

Statements commenting this question could be read in different ways: 2

One physician stated this happens more often when a patient has to pay themselves

One physician stated to take cost into consideration when it concerns ease of use rather than effectiveness

One physician remarked some subspecialisations are more prone than others (e.g. Oncology)

**Item H**

*Cost should not play a role in choosing a course of treatment*

Clarifications: 25

Some clarifications contained multiple comments.

Statements emphasising the need for rationing decisions to be made by physicians on another level (hospital policy, national policy): 7

Statements emphasising the need for rationing decisions to be made by other stakeholders (policymakers, government): 6

Statements acknowledging cost plays a role, but physicians would rather have it not play a role: 5

Statements emphasising it can only play a role when acceptable alternatives are available: 4

Statements emphasising other factors that are also important in treatment choice: 4

Statements emphasising the financial means are not endless: 4

Statements emphasising the need for the pharmaceutical industry to lower their prices: 2

Statements emphasising crowding out effect of expensive treatment: 2

One physician stated it would be very naive to agree

**Final comments**

Total additional comments: 17

Statements emphasising the need for rationing decisions to be made by other stakeholders (policymakers, government): 4

Statement regarding survey technicalities: 3

Statement proposing cost-saving measures: 2

Statement emphasising judicial consequences of rationing: 1

Statements emphasising the different degrees of ‘more effective’ and ‘more expensive: 4

Statements emphasising a lack of knowledge and awareness on healthcare cost: 3

Statement emphasising primary responsibility is individual patient: 2

Statements emphasising diagnostics is equally important in cost-containment as treatment: 2

One physician remarked the cost-consciousness of patients is virtually zero, complicating matters.

One physician remarked that the research focus possibly is too narrow, other factors such as side effects, life expectancy, age or comorbidity are also important

One physician asked to write a manifesto on healthcare cost for residents and medical students to educate them on this matter
